# Neuromuscular organization and aminergic modulation of contractions in the *Drosophila *ovary

**DOI:** 10.1186/1741-7007-4-17

**Published:** 2006-06-12

**Authors:** C Adam Middleton, Upendra Nongthomba, Katherine Parry, Sean T Sweeney, John C Sparrow, Christopher JH Elliott

**Affiliations:** 1 Department of Biology, University of York, York YO10 5YW, UK; 2 Department of Molecular Reproduction, Development and Genetics, Indian Institute of Science, Bangalore, India; 3 Centre for Neuroscience Research, Division of Veterinary Biomedical Sciences, Royal (Dick) School of Veterinary Studies, The University of Edinburgh, Summerhall, Edinburgh, EH9 1QH, UK

## Abstract

**Background:**

The processes by which eggs develop in the insect ovary are well characterized. Despite a large number of *Drosophila *mutants that cannot lay eggs, the way that the egg is moved along the reproductive tract from ovary to uterus is less well understood. We remedy this with an integrative study on the reproductive tract muscles (anatomy, innervation, contractions, aminergic modulation) in female flies.

**Results:**

Each ovary, consisting of 15–20 ovarioles, is surrounded by a contractile meshwork, the peritoneal sheath. Individual ovarioles are contained within a contractile epithelial sheath. Both sheaths contain striated muscle fibres. The oviduct and uterine walls contain a circular striated muscle layer. No longitudinal muscle fibres are seen.

Neurons that innervate the peritoneal sheath and lateral oviduct have many varicosities and terminate in swellings just outside the muscles of the peritoneal sheath. They all express tyrosine decarboxylase (required for tyramine and octopamine synthesis) and *Drosophila *vesicular monoamine transporter (DVMAT). No fibres innervate the ovarioles. The common oviduct and uterus are innervated by two classes of neurons, one with similar morphology to those of the peritoneal sheath and another with repeated branches and axon endings similar to type I neuromuscular junctions.

In isolated genital tracts from 3- and 7-day old flies, each ovariole contracts irregularly (12.5 ± 6.4 contractions/minute; mean ± 95% confidence interval). Peritoneal sheath contractions (5.7 ± 1.6 contractions/minute) move over the ovary, from tip to base or *vice versa*, propagating down the oviduct. Rhythmical spermathecal rotations (1.5 ± 0.29 contractions/minute) also occur. Each genital tract organ exhibits its own endogenous myogenic rhythm.

The amplitude of contractions of the peritoneal sheath increase in octopamine (100 nM, 81% *P *< 0.02) but 1 μM tyramine has no effect. Neither affects the frequency of peritoneal sheath contractions.

**Conclusion:**

The muscle fibres of the reproductive tract are circular and have complex bursting myogenic rhythms under octopaminergic neuromodulation. We propose a new model of tissue-specific actions of octopamine, in which strengthening of peritoneal sheath contractions, coupled with relaxation of the oviduct, eases ovulation. This model accounts for reduced ovulation in flies with mutations in the octopaminergic system.

## Background

The amines octopamine and tyramine are apparently ubiquitous in arthropods, with neuromodulatory roles in a wide range of behaviours including jumping, flight, courtship and even social interactions (see [1,2] for reviews). Although cellular actions of these neuromodulators have been described, in both the CNS and PNS, their exact roles in behaviour have resisted pharmacological approaches largely because of cross-talk between members of the amine receptor family.

Recently, the sequenced *Drosophila *genome, together with genetic knockouts of synthetic enzymes or receptors, have provided new tools to address the roles of these neuromodulators. These include two fly mutants in which the neurogenic amines octopamine or tyramine are not synthesized [3,4] and others in which one class of octopamine or tyramine receptors is deficient [5,6]. Surprisingly, these knockouts survive and appeared to behave "normally", but when fly behaviour was examined in detail, jumping ability was reduced by one-third [7] and egg-laying performance was dramatically reduced, if not abolished, suggesting that a detailed understanding of the neuromuscular basis of egg-laying would provide an excellent system for gaining an understanding of amine action.

The neuromuscular organization of egg-laying in *Drosophila *and its neuro-hormonal regulation remain poorly understood. This is surprising since many mutant lines are known in which egg-laying is reduced. Further, it is clear that the female fly controls oviposition to ensure the eggs are laid on a suitable substrate [8]. We are now able to advance this as we show below that the isolated *Drosophila *female reproductive tract maintains activity *in vitro*. This allows us to separate out aminergic modulation on the reproductive tract musculature from aminergic effects on the CNS, in ways not previously possible.

**Figure 1:**
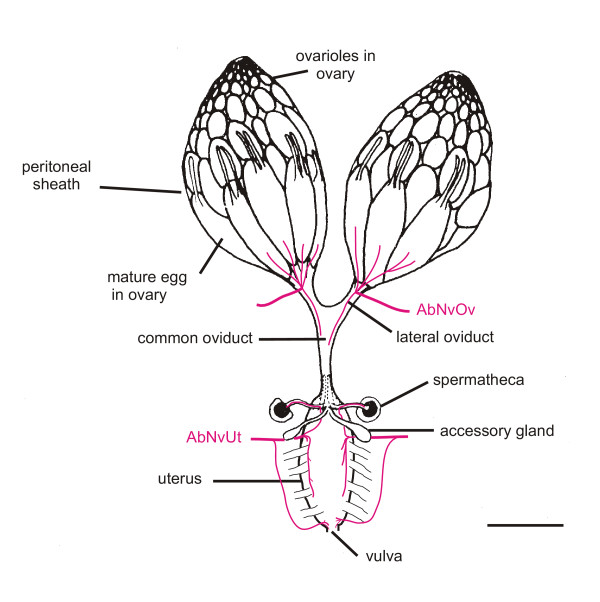
Overview of the female reproductive tract. The innervation is outlined (magenta) from our anti-HRP staining, the muscles redrawn after [49], with only a representative set of the external uterine muscles included. The two pairs of nerves, AbNvOv (abdominal nerve to the ovary) and AbNvUt (abdominal nerve to the uterus) branch from the 5^th ^pair of nerves emerging from the abdominal region of the CNS [13]. Scalebar: 250 μm approximately.

The *Drosophila *female reproductive tract is shown in Fig. 1. The eggs are formed by the ovarioles, which compose the ovary. At the tip of each ovariole is a germarium: this divides repeatedly to give a series of egg chambers, containing 15 nurse cells, an oocyte and follicle cells. As the egg chambers progress down the ovariole, they enlarge. In the final stages the nurse cell contents are transferred to the developing oocyte, the nurse cells die and the chorion is laid down, leaving a mature oocyte. Development of the oocyte is then paused until it is released from the ovary into the lateral oviduct (ovulation). It then moves into the common oviduct and into the uterus. Here, if the female has mated, the egg will be fertilised. Finally, it is oviposited on to the substrate.

**Figure 2:**
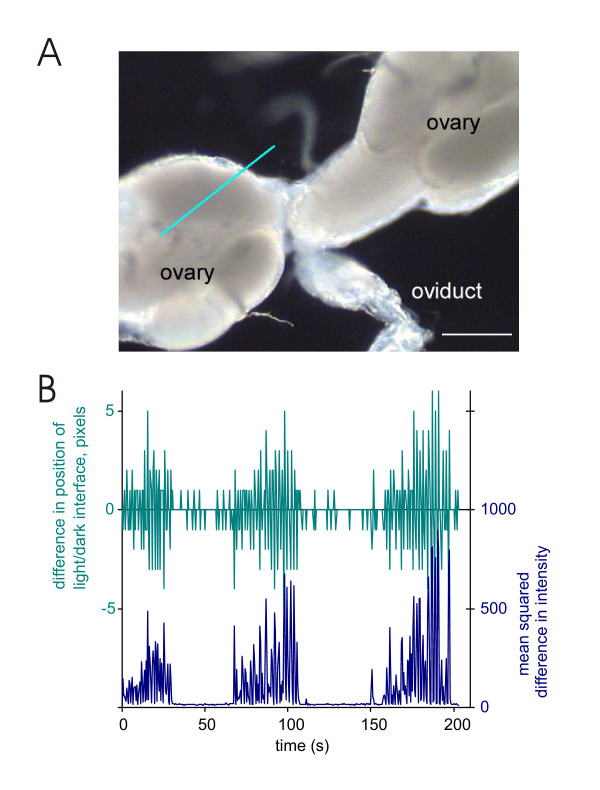
Ovariogram methodology. A. Ovariograms were constructed from the video micrographs as follows: a line was overlaid on the video image to cross the light/dark interface, in this case across the edge of the ovary. B. A plot of the change in position of the light dark boundary from frame to frame plotted (upper trace, 1 pixel is 1.7 μm) and of the mean square difference in intensity at each point along the line for successive pairs of frames (lower trace). The second method, recording the mean square difference in intensity provides a much greater sensitivity and also better discrimination of movement, as it takes into consideration the change in every pixel along the line, rather than just the position of the light/dark interface. Scalebar: 500 μm.

We have used an integrative approach to examine the neuromuscular organisation of the female genital tract, describing each muscle layer and their patterns of contraction. We show that the peritoneal sheath around the ovary and upper reaches of the oviduct are innervated by modulatory neurons, with more conventional synapses occurring in the common oviduct and uterus, but the epithelial sheaths of the individual ovarioles are not. We have developed a novel technique to record ovariograms (Fig. 2), and use this to show that each organ in the genital tract appears to have its own endogenous myogenic rhythm, with the amplitude of ovarian contractions increased by octopamine.

## Results

### Muscle structure

**Figure 3:**
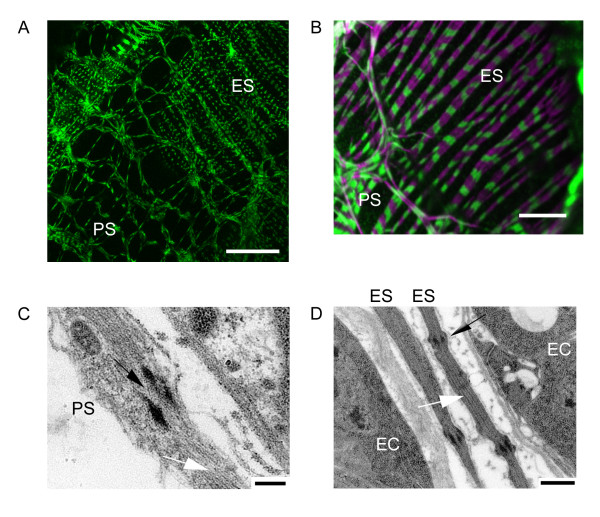
Muscle fibres of the ovary. A,B. Confocal fluorescent microscopy showing the muscle fibres of the ovariolar epithelial sheath, ES, and the ovarian peritoneal sheath, PS. The peritoneal sheath fibres overlie those of the epithelial sheath. In A, myosin (green) is *Mhc-weeP26-GFP*. In B, FITC-labelled anti-myosin antibody is green, with F-actin (magenta) detected with rhodamine-labelled phalloidin. Note that the ovariolar muscle fibres all circle the ovariole, whereas the peritoneal sheath muscle fibres form a meshwork across the ovarial surface. C, D. Electron micrographs of the peritoneal sheath (PS) and epithelial sheath (ES) respectively, showing longitudinal sections of sarcomeres with interdigitating thick and thin filaments (white arrow) and perforated Z-discs (black arrow). EC: egg chamber. Scalebars: A: 50 μm; B: 10 μm, C:200 nm, D:500 nm.

**Figure 4:**
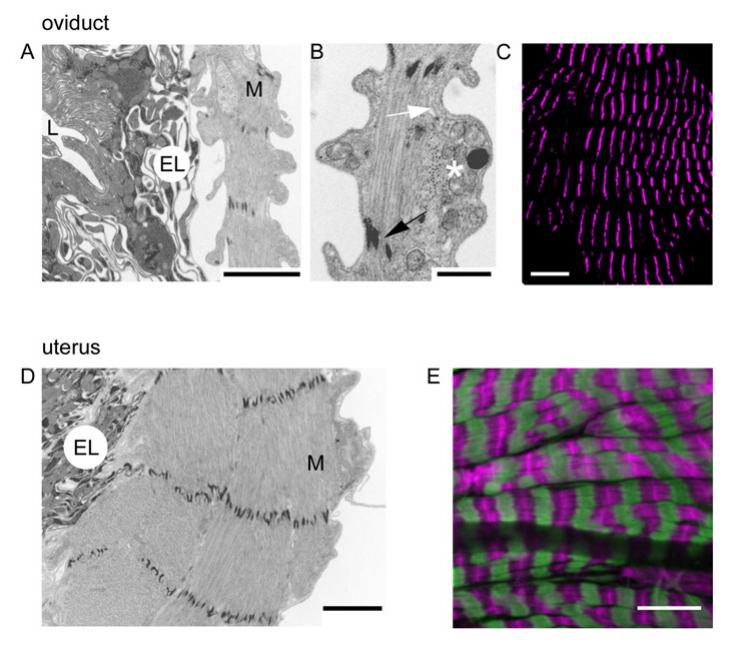
Muscles of the oviduct and uterus. A. Electron micrograph of a cross section of the common oviduct to show the outer muscular layer (M), surrounding the epithelial lining (EL) and the oviduct lumen (L). The epithelial lining has an apical surface rich in convoluted multimembrane structures. B. Detail of the muscle layer shows a myofibril with interdigitated thick and thin filaments (white arrow) and perforated Z-discs (black arrow). Occasionally some thick-thin filament lattice (asterisk) is seen cut transversely suggesting that not all the myofilaments run in parallel with the circular muscle layer, though these exceptions may be tangentially cut. C. A confocal image of a region of common oviduct with F-actin filaments stained (magenta) with rhodamine-phalloidin. This shows that the common oviduct is covered by circular muscle fibres (also seen in lateral oviducts, not shown); there is no evidence of longitudinal muscle fibres. Note that some of the phalloidin bands twist and a few split, and this may account for the transversely cut filaments seen in B. D. Electron micrograph of a uterus cross-section shows the muscle (M) and epithelial layers (EL). The muscle layer is considerably thicker than that of the oviduct and again exhibits perforated Z-discs. E. Confocal fluorescence image of uterine muscle, immunostained with myosin FITC-labelled antibody (green) and F-actin labelled with rhodamine-phalloidin (magenta) shows that the myofibrils within these cells seem to form side-by-side associations, often in partial register. The fibres have tapered ends. All the myofibres encircle the uterus; no longitudinal myofibres were seen. Scalebars: A: 2 μm, B: 0.5 μm, C: 20 μm, D: 2 μm, E: 10 μm.

The histology and ultrastructure of the major components of the female reproductive tract in *Drosophila *(the ovaries, ovarioles, lateral and common oviducts and the uterus) have been outlined previously [9,10] and here we provide particulars of the musculature. Throughout the tract, the muscle fibres are striated (see Figs. 3, 4) with a clearly defined sarcomeric structure, typical of visceral muscles in insects [11]. There is no evidence to support a claim [9] that the epithelial and peritoneal sheath fibres are 'smooth' (presumably by analogy to vertebrate smooth muscle).

Each ovariole is surrounded by an epithelial sheath (Fig. 3A,B, first described in *Drosophila *[9], that contains bands of circular muscles that run around the ovarioles. There are no muscle fibres running parallel to the long axis of the ovarioles.

In *Drosophila*, the ovarioles are held together to form two ovaries by an enveloping peritoneal sheath that is a network of muscle fibres (Fig. 3A,B) forming an open mesh around the ovary. Scanning electron micrographs [10] show the presence of large gaps (evident in Fig. 3A) in the peritoneal sheath below which the epithelial sheaths of the ovarioles can be seen.

EM sections cut longitudinally to myofibres of the peritoneal sheath (Fig. 3C) or of the two neighbouring epithelial sheaths (Fig. 3D) show the rather irregular interdigitation of the thick and thin filaments and the presence of perforated Z-discs. These are both characteristic of muscles that are supercontractile (i.e. they contract to less than 50% of their resting length when the thick filaments go through the Z-discs [11]). Supercontractile muscles occur in the insect viscera and in the larval body wall musculature [11]. This is the first indication that these peritoneal sheath muscle fibres may be supercontractile, but this is not surprising given the large volume changes that occur during post-eclosional maturation of the ovary and oogenesis.

The muscles in the walls of the common (Fig. 4A–C) and lateral oviducts (not shown) are indistinguishable. Confocal fluorescence microscopy (Fig. 4C) shows that they are striated muscles consisting of an ordered array of circular myofibres forming an almost continuous sheet around their respective lumens. Occasional twists and splits are seen in the pattern, especially around the dark "holes" that may contain the nuclei. Ultrastructurally (Fig. 4A,B) the oviduct myofibrils are significantly thicker in cross section than those in the peritoneal and epithelial sheaths around the ovariole, but are structurally identical (Fig. 3). The perforated Z-discs are very clear (Fig. 4B). Infrequently isolated areas are seen in which thick and thin filaments (myofilaments) are cut in cross-section. This may reflect the observation that not all the myofibrils within a fibre show exactly the same orientation (Fig. 4C). Although myofilaments may occasionally deviate from the fibre's long axis, the overall structure shown by confocal fluorescence microscopy did not reveal any fibres or myofibrils running longitudinally in the wall of the oviduct. The oviduct lumen (Fig. 4A) is lined by an epithelial layer (EL) that shows convoluted intracellular membranous structures and extensive microvilli on the apical surface. It seems likely that these are involved in the transport of ions and various molecules [12] to produce oviduct secretions that facilitate egg movement. The epithelial layer has separated from the muscle layer in this sample, which may indicate that the two layers are not strongly adherent *in vivo*. The circular myofibrils and muscle layer of the uterus (Fig 4D,E) are much more substantial than those of the oviducts and there is no evidence of longitudinal myofibres. Within a single myofibre, neighbouring myofibrils often appear to be in approximate register (Fig. 4E) and the myofibres seem to have tapered ends. It is not known whether these represent attachment sites through which the muscle forces are applied to the uterus or whether this occurs along the length of the myofibres. Ultrastructural studies show (Fig 4D) the sarcomeric structure of the myofibrils, again with perforated Z-discs. The muscle layer is exterior to the epithelial lining of the uterine lumen. The cells in the uterine epithelium, like those of the oviduct (Fig 4A), show a considerable amount of convoluted membranous structures consistent with secretory activity.

In all the regions of the female reproductive tract examined, we found no evidence for longitudinal muscle fibres, though occasional twisting, oblique or longitudinal myo *fibrils *were seen in the oviduct. Our confocal and ultrastructural observations show that the fibres are striated and their myofibrils have perforated Z-discs indicating that all the muscles are supercontractile [11].

### Innervation

**Figure 5:**
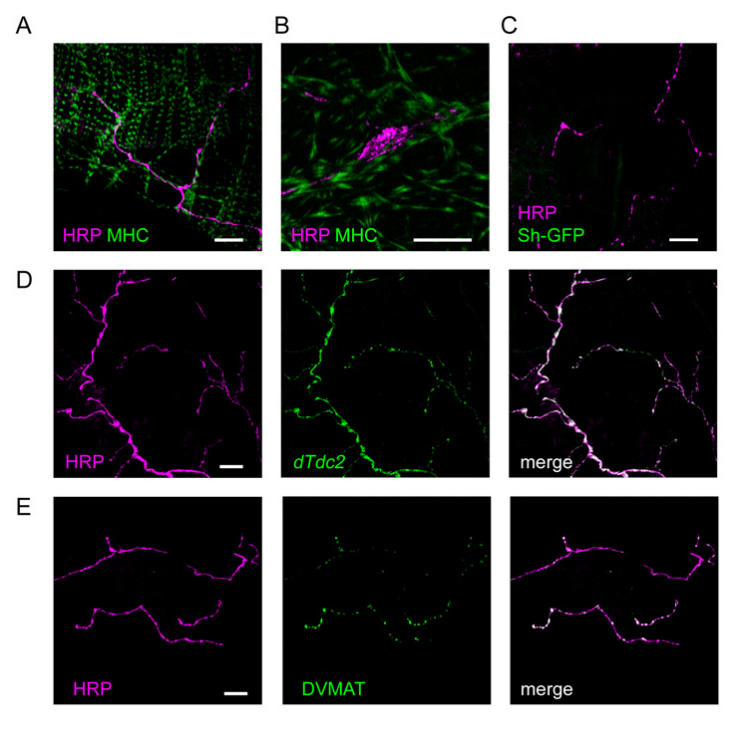
Innervation of the ovary by a single type of aminergic fibre. A. Merged confocal images from a Wee-P26 fly of nerves immunostained with anti-HRP (magenta) and of muscle expressing MHC-GFP (green) show that the nerves run along the muscle fibres of the peritoneal sheath with varicosities at irregular intervals and at their ends. The thinner muscle fibres running vertically are part of the epithelial sheath around an ovariole. B. A similar image showing a single neural varicosity at higher magnification. C. Merged confocal images showing nerves (magenta) immunostained as above in a fly expressing CD8-GFP-Shaker (green). The absence of detectable green fluorescence indicates a lack of Type I neuromuscular junctions. D. Individual and merged confocal images showing that the expression of *dTdc2-GAL4 *as reported by UAS-n-syb-spH (green) co-localising (white) with nerve fibres immunostained as above (magenta). E. Individual and merged confocal images of a specimen double-labelled with anti-HRP (magenta) and an antibody to the *Drosophila *vesicular monoamine transporter (DVMAT, green), shows that the DVMAT staining is co-incident with neural varicosities and nerve endings with occasional smaller hot-spots along the length of the nerves. No DVMAT signal was detected in the muscle fibres. Scalebars: All 20 μm.

The female genital tract is bilaterally innervated by two pairs of nerves that branch separately from the abdominal median nerve trunk (AbNvTr) [13]. One nerve projects to the junction of the ovaries and lateral oviduct (AbNvOv, Fig. 1), where it branches repeatedly. From here the nerves radiate anteriorly across the surface of the peritoneal sheath [4]. Fig. 5 shows the nerve fibres have a wandering appearance as they follow the peritoneal sheath muscle fibres. They terminate on the peritoneal sheath, mostly in the posterior two-thirds of the ovary. Confocal Z-stacks show the nerves remain outside the peritoneal sheath muscle layer; they do not dive into the gaps in the peritoneal sheath or contact the ovariolar epithelial sheath. Varicosities are observed at the ends of the nerves, and frequently also along their branches (Fig. 5B). These were often oval and did not look like the "strings of pearls" typical of type I neuromuscular junctions seen on larval or adult body wall muscles [14,15], and no glutamate receptor immunoreactivity was seen. The *Shaker *gene product (a post-synaptic potassium channel) was not detected on the surface of the peritoneal sheath or the ovarioles (Fig. 5C), although myosin was expressed in both. As the *Shaker*-GFP signal was driven with a *Mhc *driver, this suggests that the nerves do not construct Type I boutons. All the nerves on the surface of the peritoneal sheath show GFP fluorescence driven by the *dTdc2 *driver (Fig. 5D). The *dTdc2 *gene normally expresses the neuron-specific tyrosine decarboxylase. We did not find any HRP immunoreactive fibres without also detecting *dTdc2 *driven GFP fluorescence. The nerve endings and varicosities all stain brightly with antisera to the *Drosophila *vesicular monoamine transporter (DVMAT) with some staining visible between varicosities (Fig. 5E). The AbNvOv also innervates the lateral oviduct and the upper portion of the common oviduct. The lateral oviduct has the same type of innervation as the ovary, with fibres showing a similar pattern of varicosities and large endings with the same cytochemistry.

**Figure 6:**
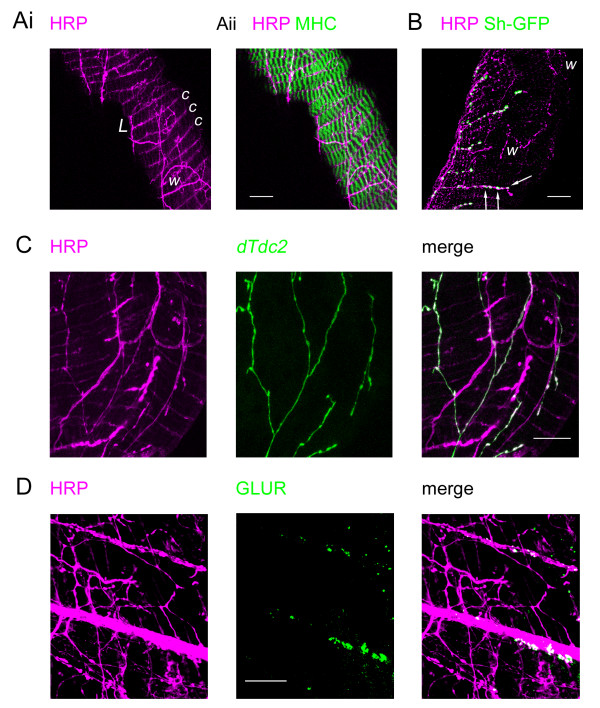
Innervation of the oviduct by two types of fibre. Ai. Confocal image from a Wee-P26 fly of nerves immunostained with anti-HRP (magenta) showing nerves running over the surface of the oviduct. Some nerve fibres run longitudinally (L) giving off circumferential branches (c) while others have a more random wandering path (w). Aii. The previous image merged with one of muscle expressing MHC-GFP shows that the circumferential branches run between the circular myofibrils. B. Merged confocal images of the oviduct from the same preparation as Figure 5C. The nerves (magenta) are immunostained as above in a fly expressing CD8-GFP-Shaker (green). Patches of CD8-Shaker fluorescence co-localise (white) with neural boutons (arrows) on the circumferential nerve fibres but are absent from the varicosities on the wandering fibres (w). C. Individual and merged confocal images showing that the expression of *dTdc2-GAL4 *as reported by UAS-n-syb-spH (green) colocalises (white) with the anti-HRP labelling (magenta) of some nerves but is absent from others. D. Individual and merged confocal images of anti-HRP labelling (magenta) and anti glutamate receptor (GLUR, green). Note that the round patches of GLUR signal are associated with the straight fibres rather than the wandering fibres. Scalebars: All 20 μm.

On the common oviduct, a dual pattern of innervation is present. Wandering fibres (Fig. 6A,B) similar to those on the peritoneal sheath and lateral oviduct are present and they co-localise with the *dTdc2 *gene marker (Fig. 6C), but show no glutamate receptor staining (Fig. 6D). The second type of fibre often runs along the common oviduct (Fig. 6A), and has branches running circularly parallel to the myofibres (Fig. 6A, marked c), (though it is possible that some of the finer parts result from immunoreaction with the muscle cell surface). These neurons do not show *dTdc2 *driven GFP fluorescence or anti-DVMAT immunostaining, or have the oval varicosities, but rather are associated with round blebs (Fig. 6B). Many of these blebs are correlated with GFP-label from the Shaker potassium channel construct, though this fluorescence is in a focal plane closer to the muscle layer. These blebs are also associated with glutamate receptor immunoreactivity (Fig. 6D).

The extrinsic and intrinsic muscles of the uterus are innervated by a second pair of nerves, branching from the abdominal median nerve trunk. This pair of nerves (AbNvUt, Fig. 1) innervates both the extrinsic muscles and the circular myofibres (data not shown). These nerves have small boutons, closely apposed to larger, round blebs of Shaker-fluorescence, suggesting type I-like terminals. One branch projects to the inner layers of the uterus and shows our *dTdc2 *marker.

### Ovariograms

**Figure 7:**
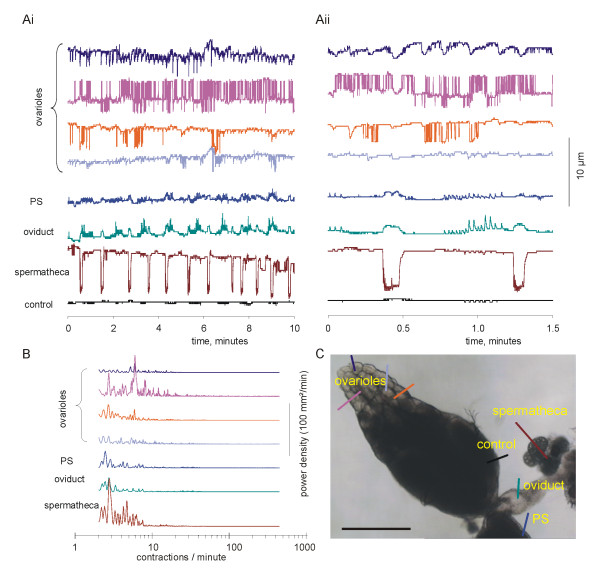
Independent movements in the female *Drosophila *reproductive tract. Ai. Simultaneous recording from 4 ovarioles, the peritoneal sheath (PS), oviduct and spermatheca, plus a stationary control. In this preparation, the bases of the two ovaries and uterus lie on the microscope slide – the spermatheca and oviducts are free to move within the saline, while the ovarioles contract within the ovary. Video recorded at 15 frames/second; only the position of the light/dark boundary is shown. Aii. Part of the record is shown expanded. Note that the spermatheca has the largest movement, when its duct uncurls for 5–6 seconds at a time. The movement in the spermatheca has a small, gradual pull on the PS and oviduct, but small, higher frequency waves are also seen in these organs. The oviduct trace shows that its contractions occur at random with respect to those of the spermatheca, sometimes occurring before, at other times during or after those of the spermatheca. Each of the ovarioles has independent activity within the ovary, ranging from fast and frequent to occasional and erratic. Note that, at the PS recording site (see Fig. 7C), the PS can be seen as a lighter colour fringe, while on the other ovary control recordings were taken where the peritoneal sheath is stretched tautly over the eggs and so no PS waves were apparent. B. Fast Fourier transforms of the data shown in A, plotting the power density from 2 to 300 contractions/minute. Although the raw FFT output extended below 2 contractions/minute, this corresponds to slow drift in the preparation rather than biological activity. Virtually no energy occurs above 60 contractions/minute (1 Hz), so recording at 2 frames/second captures the movement faithfully. Note that the ovarioles all have peaks at different frequencies. For the spermatheca, the nearly square waveform means that the main peak is accompanied by several side bands. C. The first frame of the video, showing the position at which the recordings were taken. The video is available at reduced sample rate in QuickTime format [see Additional file 1] or in other formats [50]. Scalebar: 250 μm.

Ovariograms were made by digital video microscopy of the isolated reproductive tract placed on a microscope slide. Data were analysed by overlaying lines on the computer image, and the movement of the light/dark interface along the line was determined (Fig. 2). Fig. 7 shows the analysis of contractions of 4 ovarioles, the peritoneal sheath (PS), oviduct and a spermatheca. The ovariograms consistently show that the isolated genital tract is spontaneously active. The ovarioles, peritoneal sheath, oviduct and spermathecae contract rhythmically and for the most part independently. Oviduct contractions are often associated with those of the peritoneal sheath; they last 1–2 seconds and have a rapid rise and fall. However, oviduct movements, rather than contractions, are also correlated with that of the spermatheca, owing to their strong mechanical coupling. These are much more gradual and last for 10–15 seconds.

Each ovariole moves independently within the peritoneal sheath, with the mean rate being 12.5 ± 6.4 contractions/minute (mean ± 95% confidence interval; mode 10 contractions/minute; 17 ovarioles measured in 6 preparations). There is a very diverse pattern of contractions: some move almost continuously, with up to 53 contractions/minute (Fig. 7A pink trace), others with regular bouts of activity and others remaining largely quiescent (light blue trace, 5 of the 17 ovarioles showed less than 2 contractions/minute). As shown by the traces in Fig. 7(A,B), different ovarioles with a single ovary behave quite differently

The largest recorded movements are those of the spermathecae. These are on the termini of a long thin duct, which contracts or rotates the spermatheca, so that its movements are always very large. In 7-day-old flies these show regular contractions (1.5 ± 0.29/minute, N = 6) with individual contractions lasting 5–6s.

**Figure 8:**
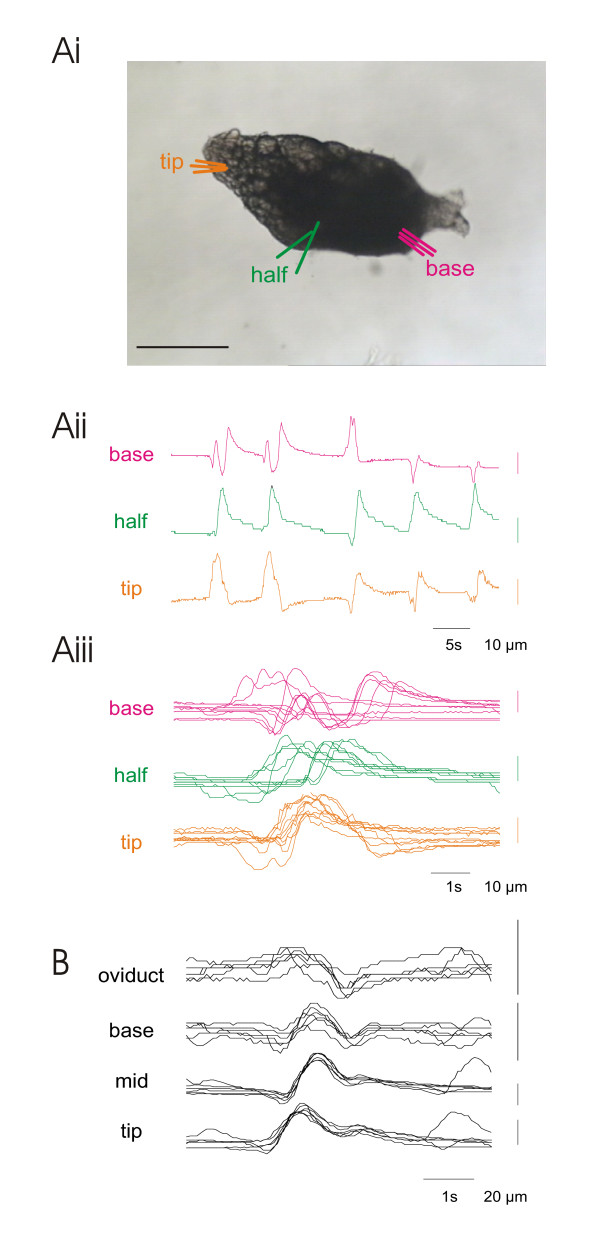
Irregular wavepaths for contractions of the isolated ovary. Ai. Replicate measures of the position of the light/dark interface were made at three sites; the same colour is used for each site. For each site, three adjacent replicates were made and the signal averaged to reduce the noise. Aii. The resulting ovariogram shows that several kinds of contractions were observed, some as a single peak, others biphasic and some triphasic. Aiii. Overlaying the recordings so that waves are aligned at the tip shows that some waves move from tip to base, others from base to tip. This was observed in both the isolated ovary and the reproductive tract complete. B. Similar recordings from another preparation, showing consistent waves of contraction moving from tip to base and on into the oviduct. Scalebar: 250 μm.

Contractions of the peritoneal sheath are largest at the base of the ovary and so most easily recorded here. In 7-day-old flies the mean frequency of contractions was 5.7 ± 1.6/minute (in preparations in which the spermatheca and seminal receptacle and uterus had been removed to avoid mechanical coupling). In a few flies, we observed eggs leaving the ovary; the progress of the egg was correlated with bursts of contraction. The signal/noise ratio for the peritoneal sheath contractions is quite low in 7-day-old flies, when measuring the position of the light/dark boundary. Using the mean square intensity method gives good clear recordings of the peritoneal sheath waveform (Fig. 2), while in 3-day-old flies, the amplitude of contractions is bigger than in 7-day-old flies because the eggs are less developed and so the sheath is less taut. Consequently the signal/noise ratio is better and the waves are clear to see (Fig. 8). We isolated ovaries from 3-day-old flies and used their ovariograms (Fig. 8) to investigate the regularity of the peritoneal sheath contractions. In at least half the preparations, wavepaths across the peritoneal sheath vary, with some waves having a single contraction peak and others multiple peaks (Fig. 8 Aii). By overlaying each wave, aligning the peaks for the tip, we found that some waves occurred at the tip before the base, while other moved from base to tip, and others had a complex biphasic wavepath (Fig. 8iii). In other preparations, we observed a consistent pattern, for example in Fig. 8B, where the wave of contraction consistently moved from the tip to the base.

**Figure 9:**
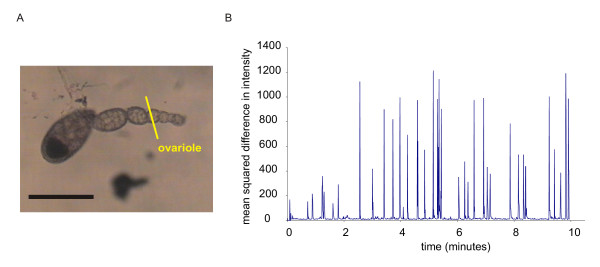
An individual ovariole continues to contract in isolation. A. The first frame of the video, showing the location of the recording site. B. Contractions are monitored by changes in the mean squared difference in intensity between successive frames. Data recorded at 2 frames/second. Scalebar: 100 μm.

Individual ovarioles teased from ovaries still contract spontaneously. Each has its own pattern of bursts, with waves moving both backwards or forwards along the ovariolar epithelial sheath (Fig. 9). This is unlike the observations reported from *Drosophila hydei *where waves always moved from the base (pedicel) to the tip of the ovariole [16]. The mean contraction frequency of isolated ovarioles, 2.9 ± 1.0 contractions/minute is about one third of their activity within the intact ovary.

The Fast Fourier Transform (FFT, Fig. 7B) reveals that the spermathecae have the greatest power density at 2.8 contractions/minute, owing to their freedom to move with large amplitude as their ducts contract and rotate. The spermatheca FFT has prominent sidebands, as would be expected from their tendency to remain contracted for 5–6s. The oviduct (and the peritoneal sheath) share many peaks with the spermatheca, as they are tightly mechanically coupled in this isolated preparation. The peritoneal sheath contractions show a series of small peaks in the spectrum from 10 to 40 contractions/minute, reflecting endogenous peritoneal sheath contractions, some shared with the oviduct. The ovarioles have a different peak power density, in the range 3 to 16 contractions/minute.

### Pharmacology

We have begun our pharmacological analysis by examining the response of the peritoneal sheath to octopamine and tyramine because the contractions of the peritoneal sheath are an essential first step in the movement of the egg along the reproductive tract. We used the ovary and oviduct preparation to avoid contamination of the traces by spermathecal or uterine contractions. The mean duration of the samples was 145 ± 8 s.

**Figure 10:**
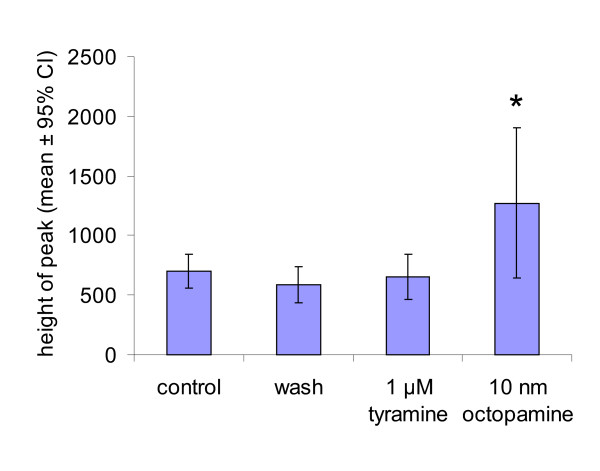
Octopamine, but not tyramine, increases the amplitude of peritoneal sheath contractions. Data from preparations in which the uterus and spermathecae were removed to avoid mechanical coupling. Data recorded as mean square difference in intensity, with the average height of a peak calculated for each preparation. Control: N = 41 peaks; wash N = 27; 1 μM tyramine N = 10; 10 nM octopamine N = 7. Unpaired t-tests were used to compare drug treatments, with * used to indicate P < 0.05.

Our ovariograms from 7-day-old flies show that the amplitude of the peritoneal sheath contractions at the base of the ovary increases with 100 nM octopamine (81% increase, P < 0.02, N = 6) (Fig. 10). In contrast, tyramine application at doses up to 1 μM has no significant effect on the amplitude (P = 0.77, N = 12). After application of the neuromodulator, preparations were washed and the contraction amplitudes measured to be 83% of the control value (P = 0.3, N = 31).

**Figure 11:**
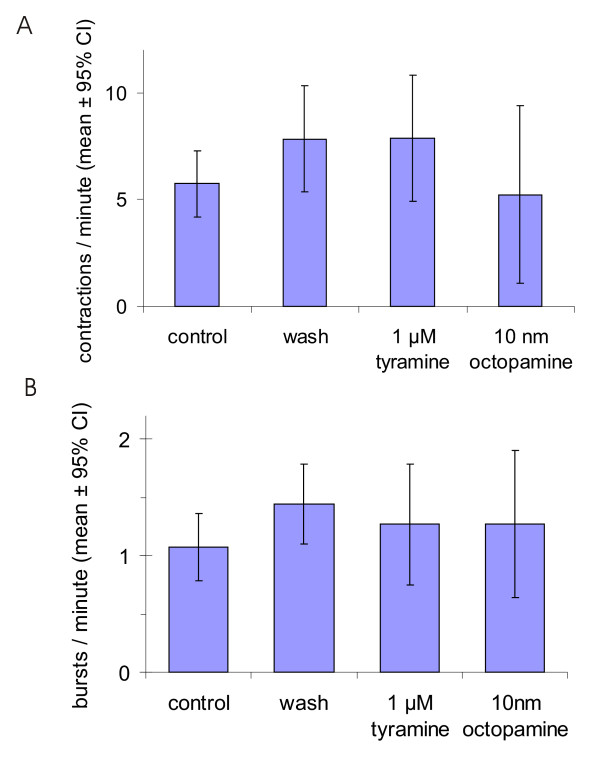
There is no statistically significant change in either (A) the frequency of contraction or (B) the frequency of bursts of contractions in the peritoneal sheath with octopamine or tyramine. Same preparations and analysis as Fig. 10.

The mean frequency of peritoneal sheath contractions from 7-day-old flies (5.7 ± 1.6 contractions/min) did not change significantly following application of octopamine or tyramine, either by comparison of all preparations or by paired comparison of individual preparations (Fig. 11A). Contractions typically occur in bouts, which occur every 0.3–2.5 minutes. We used a minimum interburst interval of 0.2 minutes and determined the mean frequency of bouts to be 1.1 ± 0.28 bouts/minute. The occurrence of bouts was also not affected by either of these drugs (Fig. 11B).

## Discussion

### Muscle movements of the genital tract

The female reproductive tract is a muscular system, in which contractions are linked to movement of the eggs down the tract from ovarioles via the oviducts to the uterus and finally oviposition of the fertilised eggs. We have described circular fibres in the muscle layers of tubular tissues of the *Drosophila *reproductive tract (ovarioles, oviduct and uterus). In the walls of the larval and adult gut and in some other insect duct systems, the layers of circular muscles are frequently opposed by longitudinal muscles [11]. Longitudinal fibres were apparently detected during development of the *Drosophila *ovary [9], and have been found, along with circular myofibres, in the ovaries of a number of Lepidoptera including the silk moth *Hyalophora cecropia *[17], the flour moth *Ephestia kühniella *[18], the sugar cane borer *Diatraea saccharalis *[19] and the butterfly *Calpodes ethlius *[20]. However, we have found no evidence for the presence of longitudinal fibres in the female *Drosophila *genital tract using electron microscopy, immunocytochemistry or directed GFP expression. This is consistent with the movements measured in our ovariograms, where peristaltic waves, but not shortening, were observed. The *Drosophila *ovary is covered by a peritoneal sheath, which as noted by others [9,10] contains a network of muscle fibres. Similar sheaths have been reported from other Diptera [21] and may perform a similar function to that of the longitudinal fibres described in other insects; the Lepidopteran ovaries lack peritoneal sheaths. The neural co-ordination of muscle contractions in the *Drosophila *peritoneal sheath and various tubular muscles are likely to be important for egg development and oviposition. These contractions may also be important for moving the haemolymph around the reproductive tract, especially within the ovary during the energetically and nutritionally demanding process of oogenesis.

Our anti-HRP immunostaining showed that the nerves to the ovary run along the peritoneal sheath network, outside the muscle layer rather than among its myofibrils. We have detected no direct innervation of the myofibres of the ovariolar epithelial sheath. Thus it seems likely that the contractions of the ovariole sheath are controlled by a (local) hormonal mechanism. This would fit with the persistence of spontaneous myogenic contractile activity in the isolated ovariole.

The peritoneal sheath and lateral oviduct have a common pattern of innervation, in which the nerve fibres make oval varicosities, both along their length and at their endings. This is much closer to the type II neuromuscular junction boutons described in adult thoracic muscles [15] than to the type I. We propose that they are modulatory terminals: a suggestion supported by the lack of the Shaker channels, which are usually seen opposite type I terminals [22], as is glutamate receptor immunostaining. The lack of Shaker and glutamate fluorescence was not due to poor staining as the common oviduct and uterus of the same specimens consistently showed them at similar confocal settings. We have direct evidence that these nerves are all aminergic using two markers for tyramine and octopamine synthesising neurons. First, we used a brighter GFP to extend previous data [4], showing the presence of *dTdc2 *GFP marker on the peritoneal sheath [4] by demonstrating that this marker highlights not just some but all the nerves running over the surface of the peritoneal sheath. As tyrosine decarboxylase (TDC) is essential for the production of both tyramine and octopamine the implication is that these are all tyramine and/or octopamine releasing neurons. Secondly, we find that all the terminals are immunostained with the *Drosophila *vesicular monoamine transporter (DVMAT) antiserum [23]. There is a difference in intracellular location of *dTdc2 *GFP and DVMAT staining: the *dTdc2 *GFP co-localises with HRP marker along the length of the neurons, while the DVMAT is only seen at and between varicosities. The ovarial innervation was detected with an antiserum to tyramine β-hydroxylase, the enzyme that converts tyramine to octopamine [24]. An antiserum to the OAMB octopamine receptor shows staining in the lateral and common oviducts, in the peritoneal sheath and possibly in the ovarioles [6]. Taken together these observations further support the contention that these neurons release octopamine and or tyramine at the peritoneal sheath and our ovariograms confirm that octopamine increases peritoneal sheath contractions. Although the fibres seen by these approaches are the same as we find by anti-HRP staining, it remains possible that not all the neurons use tyramine/octopamine, and that a proportion of the neurons use other transmitters or use a co-transmitter (e.g. a peptide) to modulate the activity of the ovary. Anatomically similar endings have been described on the hindgut and shown to be proctolin-immunoreactive [25].

The common oviduct and uterus show a second pattern of innervation, where nerves, ending as round boutons, on the muscle fibre layers were common. Nerve and muscle are arranged systematically, with nerve branches running parallel to the circular muscle fibres. Here we found glutamate receptors (GluRA; though other subtypes may be present). Our detection of these receptors and of the post-synaptic Shaker channel under the blebs, both typical of adult type I neuromuscular junctions [15], coupled with synaptotagmin immunostaining of the fine endings in the muscle layer [26], suggests conventional neuromuscular junctions rather than those associated with neurohormonal release. In the oviduct, we also observed modulatory endings very similar to those found on the peritoneal sheath, in agreement with the *dTdc2 *or tyramine β-hydroxylase staining [4,24]. Modulatory endings in the luminal layers of the uterine muscle have not been reported previously.

### Ovariograms

Observations of movement in isolated ovaries has been reported previously using casual observations or videotape [16,21]. Our technique to quantify the movement of the *Drosophila *reproductive tract shows persistent, steady recordings in HL-3 saline. Discrimination of movement of different parts of the reproductive tract shows that the ovarioles, the peritoneal sheath of the ovary, oviduct and spermatheca all generate independent contractions. In each organ, waves of contraction come in bursts, separated by longer quiescent periods. These have all been recorded in isolation from the CNS and so are myogenic in origin. This is confirmed by the persistence of ovariolar rhythms in the isolated ovariole, and by the continued contraction of the peritoneal sheath in the isolated ovary. Myogenic rhythms have been recorded from a range of insect visceral tissues, including the gut and reproductive organs. In the gut, different regions display independent rhythms, where bath-applied amines and peptides modulate large changes in the amplitude and frequency of contractions [27,28].

### Pharmacology

Our data indicate that octopamine significantly increases the amplitude of the peritoneal sheath contractions. In locusts (*Locusta migratoria*), octopamine reduces the spontaneous contractions of the oviduct [29,30]. It diminishes both the frequency of the myogenic rhythm and basal tone. In addition, octopamine reduces the proctolin-induced contractions of the oviduct, with 50% block between 10^-6 ^and 10^-7 ^M. Similar data come from a dipteran, the stable fly (*Stomoxys*), in which octopamine also causes a reduction in the spontaneous and proctolin-induced contractions of the oviduct [31].

Our data have led us to a new model in which tissue-specific differences are important. Octopamine increases the strength of contractions of the peritoneal sheath, which will facilitate ovulation of the egg. At the same time, we expect octopamine to relax the oviduct, so that the egg can move into it more easily. Thus, one neuromodulator acts on two neighbouring zones with opposing effects to enhance reproductive success. This hypothesis is in accord with the locust oviduct data, and also with *Stomoxys*, where 10–100 nM octopamine increases the amplitude of ovarian contractions but relaxes the oviduct [21,31].

This model resolves a paradox. Previous authors found it hard to reconcile observations that flies lacking octopamine (owing to the *M18 *null mutation in the tyramine β-hydroxylase gene) or with a null mutation in the *Oamb *type of octopamine receptor lay fewer eggs than wild type [6,24] with the fact that octopamine reduces reproductive tract contractions. Our observations provide a simple explanation for the loss of egg-laying in octopamine-free flies: we suggest that octopamine is likely to be released during egg-laying behaviour to enhance the strength of ovarian contractions and to relax the oviduct and so speed the egg along the reproductive tract. Octopamine may also affect the spermatheca; in locusts the spermathecal contractions were increased by octopamine [32], and the oviduct relaxed [29].

Finally, we showed that tyramine had no effect on the amplitude of the contractions. This is different from the response of the locust oviduct, where tyramine had a very similar inhibitory effect to octopamine on the contraction frequency and basal tone [33]. In locust, the effect of octopamine was mediated by cAMP, but the tyramine response did not depend on a cAMP pathway except at very high (10^-4 ^M) concentrations. This suggests a difference in receptor activation.

In flies, several "octopamine" receptors have been reported. One sensitive to both tyramine and octopamine (*TyrR *= *hono*) [34,35] is unlikely to be the receptor on the peritoneal sheath, which was only sensitive to octopamine. At least four receptors (*Oamb *and the DmOctβ receptors [36-38]) are two orders of magnitude more sensitive to octopamine than tyramine, in accord with our pharmacology, and so may be present on the peritoneal sheath. Although *Oamb *has been localised on the oviduct [6], the receptor on the peritoneal sheath awaits identification.

## Conclusion

The reproductive tract of the female fly has only circular muscles and no longitudinal muscles. They are supercontractile, which is appropriate for a system that has to distend when the egg passes through. The muscles have complex, bursting myogenic rhythms, with independent oscillators in the ovarioles, in the peritoneal sheath around the ovary and in the spermatheca. The ovary is innervated solely by modulatory neurons, which are tyraminergic and/or octopaminergic. The oviduct and uterus are innervated both by similar aminergic fibres and by glutamatergic neurons with endings similar to type I neuromuscular junction. Octopamine, but not tyramine, modulates the peritoneal sheath around the ovary, increasing the strength of the contraction, and we propose it also relaxes the oviduct. This double effect eases the ovulation of the egg from the ovary to the oviduct.

## Methods

### Flies

Flies were maintained at 25°C on standard yeast-sugar-agar medium. Wild type was *Canton-S*. Some observations used flies from the Wee-P26 stock [39], which carries a green fluorescent protein (GFP) coding sequence inserted into the myosin heavy chain gene, *Mhc*. We used a stock in which the CD8-GFP-Shaker protein is driven by a *Mhc *gene promoter [22]. This expresses Shaker-GFP in the post-synaptic densities at the type I neuromuscular junctions of the nerves ending on the muscles. To visualise the synapses of neurons expressing tyrosine decarboxylase 2 (TDC2), we used the *dTdc2-GAL4 *construct [4] to drive expression of UAS-n-syb-spH, an enhanced GFP fused to neuronal synaptobrevin [40].

### Immunocytochemistry and microscopy

Reproductive tracts from 7-day old females were fixed at room temperature for 30 min in 4% paraformaldehyde in phosphate buffered saline (PBS) or for 3 minutes in Bouin's solution (Sigma-Aldrich, UK), washed twice with PBS and incubated in two changes of 0.5% Triton-X100 in PBS for 30 minutes. After further PBS washes, specimens were treated with antibodies or phalloidin-TRITC. Some treatments were applied sequentially to provide a double label. Dilutions and washes were made in PBS.

Actin was labelled by incubation in 50 nM TRITC-labelled phalloidin (Sigma-Aldrich) for 2 h at room temperature. Myosin was labelled by incubation in rabbit anti-myosin heavy chain antibody (1:1000) [41] overnight at 4°C, followed, after washing, by a further 3 h incubation at room temperature in FITC-conjugated goat anti-rabbit IgG antibody (1:250) (Sigma-Aldrich). Vesicular monoamine transporter was labelled by incubation in rabbit anti-*Drosophila *vesicular monoamine transporter antibody (anti-DVMAT-A, 1:500; [23]) overnight at 4°C followed by washing and incubation in FITC-conjugated goat-anti rabbit IgG antibody as described above. Glutamate receptors were labelled as described [42] by incubation with mouse anti-glutamate receptor (dGluRIIA, 1:5) [43], overnight at 4°C, followed, after washing, by a further 3 h incubation at room temperature in FITC-conjugated goat anti-mouse IgG antibody (1:250) (Sigma-Aldrich). Nerve fibres were labelled by incubation in Cy3-conjugated anti-horseradish peroxidase antibody (anti-HRP, 1:1000, Jackson ImmunoResearch, West Grove, PA, USA) for 2 h at room temperature.

After labelling, all specimens were washed in PBS and mounted in Vectashield (Vector Laboratories, Burlingame, CA, USA). Confocal images were obtained with a Zeiss LSM510 META mounted on a Zeiss Axioplan 2 M microscope.

### Electron microscopy

Samples of female reproductive tract were processed for electron microscopy as described for flight muscle [44].

### Ovariograms

Contractions of the isolated reproductive tract were recorded using video-microscopy followed by computer analysis of the movements from frame to frame. This approach produces an ovariogram in which the contractions at several sites on the same preparation can be recorded and compared.

Flies were transferred to vials within 24 hours of hatching and used on day 3 or day 7. Each vial contained both males and females, so our data are assumed to be from fertilised females. Complete female genital tracts were dissected into cold calcium-free HL-3 medium [45]. In most experiments, the spermathecae and seminal receptacle were removed to avoid the large mechanical coupling between spermatheca and oviduct movements (Figs. 10, 11). In some experiments oviducts were also removed to facilitate ovariogram recordings from isolated ovaries (Fig. 8).

Preparations were mounted in fresh HL-3 saline (Ca concentration 1 mM) at 22°C on cavity microscope slides for 20 minutes before observations began. Data were obtained using a Nikon Labophot microscope with a 4× objective through a 5× photographic eyepiece with a Panasonic WV-CL10 camera. The video recordings were captured directly to a 1 MHz PC equipped with a Hauppauge Win-TV capture card (44805), using the VirtualDub 1.5.3 software package and the Huffyuv lossless compression algorithm (Huffyuv Codec) [46]. Resolution was set at 640 × 480 pixels, capturing at 2 or 15 frames/second, average recording time 145 ± 8 s.

Video was analysed using a custom program (avi_line, [47]). In this, the mouse was used to overlay lines on the video frames, so that each line crossed the light/dark boundary between the preparation and the background. For each frame, the distance of the light/dark interface from the start of the line was determined, along with the mean squared difference in intensity between successive frames of the pixels along the line (Fig. 2). This records the displacement in the plane of focus, but any movement in the vertical direction is not measured. Data were saved in a Microsoft Excel format and mean peak height and intervals between peaks calculated. The Fast Fourier Transforms (FFT) were calculated by exporting the position of the light/dark interface into the fft program from Physionet [48].

In a few experiments, ovarioles from a single ovary were teased apart and video recorded as above but using a 10× phase contrast objective (Fig. 9).

### Pharmacology

After the initial 20 minute acclimatisation, the activity of the isolated reproductive tract was recorded for 2–3 minutes. The solution was then changed for one containing octopamine or tyramine and a further 2–3 minutes recorded. The control HL-3 solution was then applied for another 2–3 minutes recording. Because of disk space limitations, we were unable to record for longer, and this may affect the accuracy of our measurements of the frequency of contraction. Amines and HL-3 saline components were from Sigma-Aldrich, UK.

### Statistics

For each preparation, the mean amplitude and interval between contractions were calculated. Differences between these means were tested in Minitab, using Mann-Whitney and Student's *t*-tests. All data are reported as mean ± 95% confidence interval.

## Authors' contributions

In this joint study, JCS, CAM and UN contributed muscle studies, CAM and CJHE the innervation, CJHE and KP the ovariograms and STS the genetic reporters. CJHE, JCS, STS, UN and CAM all contributed to writing and revising the manuscript.

## Supplementary Material

Additional File 1 Video micrograph of the reproductive tract of a 7 day old female fly, showing one ovary, ovarioles, oviduct and spermatheca. During the video, note the contractions of the peritoneal sheath, ovarioles and spermatheca. This video shows 25 s of the data analysed in Fig. 7, resampled at 1.5 frames/second. QuickTime format video (created with MS-Windows v6.50; other formats of this video available online [50]). Click here for file

## References

[B1] Roeder T, Seifert M, Kahler C, Gewecke M (2003). Tyramine and octopamine: Antagonistic modulators of behavior and metabolism. Arch Insect Biochem Physiol.

[B2] Roeder T (1999). Octopamine in invertebrates. Prog Neurobiol.

[B3] Monastirioti M, Linn CE, White K (1996). Characterization of *Drosophila *tyramine beta-hydroxylase gene and isolation of mutant flies lacking octopamine. J Neurosci.

[B4] Cole SH, Carney GE, McClung CA, Willard SS, Taylor BJ, Hirsh J (2005). Two functional but noncomplementing *Drosophila *tyrosine decarboxylase genes: distinct roles for neural tyramine and octopamine in female fertility. J Biol Chem.

[B5] Kutsukake M, Komatsu A, Yamamoto D, Ishiwa-Chigusa S (2000). A tyramine receptor gene mutation causes a defective olfactory behavior in *Drosophila melanogaster*. Gene.

[B6] Lee HG, Seong CS, Kim YC, Davis RL, Han KA (2003). Octopamine receptor OAMB is required for ovulation in *Drosophila melanogaster*. Dev Biol.

[B7] Zumstein N, Forman O, Nongthomba U, Sparrow JC, Elliott CJH (2004). Distance and force production during jumping in wild type and mutant *Drosophila melanogaster*. J Exp Biol.

[B8] Grossfield J, Ashburner M, Wright TRF (1978). Non-sexual behavior of *Drosophila*. The Genetics and Biology of Drosophila.

[B9] King RC (1970). Ovarian development in Drosophila melanogaster.

[B10] Mahowald AP, Kambysellis MP, Ashburner M, Wright TRF (1980). Oogenesis. The Genetics and Biology of Drosophila vol 2d.

[B11] Crossley AC, Ashburner M, Wright TRF (1978). The morphology and development of the *Drosophila *muscular system. The Genetics and Biology of Drosophila.

[B12] Cook BJ, Pryor NW (1996). Structural characterization of peripheral nerve cells and nerve-muscle junctions of the oviduct of stable fly (Diptera: Muscidae). J med Ent.

[B13] Hertweck H (1931). Anatomie und Variabität des Nervensystems und der Sinnesorgane von *Drosophila melanogaster *(Meigen). Z wiss Zool.

[B14] Bellen HJ, Budnik V, Sullivan W, Ashburner M, Hawley RS (2000). The neuromuscular junction. Drosophila protocols.

[B15] Rivlin PK, St Clair RM, Vilinsky I, Deitcher DL (2004). Morphology and molecular organization of the adult neuromuscular junction of *Drosophila*. J Comp Neurol.

[B16] Büning J (1994). The Insect Ovary.

[B17] King RC, Agarwal SK (1965). Oogenesis in *Hyalophora cecropia*. Growth.

[B18] Cruickshank WJ (1973). The ultrastructure and functions of the ovariole sheath and tunica propria in the flour moth. J Insect Physiol.

[B19] dos Santos DC, Gregorio EA (2002). Ultrastructure of the ovariole sheath in *Diatraea saccharalis *(Lepidoptera: Pyralidae). Biocell.

[B20] Griffith CM, Lai-Fook J (1986). The ovaries and changes in their structural components at the end of vitellogenesis and during vitelline membrane formation in the butterfly, *Calpodes*. Tiss Cell.

[B21] Cook BJ, Peterson T (1989). Ovarian muscularis of the stable fly *Stomoxys calcitrans *– Its structural, motile, and pharmacological properties. Arch Insect Biochem Physiol.

[B22] Zito K, Fetter RD, Goodman CS, Isacoff EY (1997). Synaptic Clustering of Fasciclin II and Shaker: Essential Targeting Sequences and Role of Dlg. Neuron.

[B23] Greer CL, Grygoruk A, Patton DE, Ley B, Romero-Calderon R, Chang HY, Houshyar R, Bainton RJ, Diantonio A, Krantz (2005). A splice variant of the *Drosophila *vesicular monoamine transporter contains a conserved trafficking domain and functions in the storage of dopamine, serotonin, and octopamine. J Neurobiol.

[B24] Monastirioti M (2003). Distinct octopamine cell population residing in the CNS abdominal ganglion controls ovulation in *Drosophila melanogaster*. Dev Biol.

[B25] Anderson MS, Halpern ME, Keshishian H (1988). Identification of the neuropeptide transmitter proctolin in *Drosophila *larvae: characterization of muscle fiber-specific neuromuscular endings. J Neurosci.

[B26] Finley KD, Edeen PT, Foss M, Gross E, Ghbeish N, Palmer RH, Taylor BJ, McKeown M (1998). *dissatisfaction e *ncodes a tailless-like nuclear receptor expressed in a subset of CNS neurons controlling *Drosophila *sexual behavior. Neuron.

[B27] Nassel DR (1996). Peptidergic neurohormonal control systems in invertebrates. Curr Opin Neurobiol.

[B28] Hertel W, Pass G (2002). An evolutionary treatment of the morphology and physiology of circulatory organs in insects. Comp Biochem Physiol A.

[B29] Lange AB, Tsang PKC (1993). Biochemical and physiological effects of octopamine and selected octopamine agonists on the oviducts of *Locusta migratoria*. J Insect Physiol.

[B30] Nykamp DA, Lange AB (2000). Interaction between octopamine and proctolin on the oviducts of *Locusta migratoria*. J Insect Physiol.

[B31] Cook BJ, Wagner RM (1992). Some pharmacological properties of the oviduct muscularis of the stable fly, *Stomoxys calcitrans*. Comp Biochem Physiol C.

[B32] Clark J, Lange AB (2003). Octopamine modulates spermathecal muscle contractions in Locusta migratoria. J Comp Physiol A Neuroethol Sens Neural Behav Physiol.

[B33] Donini A, Lange AB (2004). Evidence for a possible neurotransmitter/neuromodulator role of tyramine on the locust oviducts. J Insect Physiol.

[B34] Saudou F, Amlaiky N, Plassat JL, Borrelli E, Hen R (1990). Cloning and characterization of a *Drosophila *tyramine receptor. EMBO J.

[B35] Reale V, Hannan F, Midgley JM, Evans PD (1997). The expression of a cloned *Drosophila *octopamine/tyramine receptor in *Xenopus *oocytes. Brain Research.

[B36] Han KA, Millar NS, Davis RL (1998). A novel octopamine receptor with preferential expression in *Drosophila *mushroom bodies. J Neurosci.

[B37] Balfanz S, Strunker T, Frings S, Baumann A (2005). A family of octapamine receptors that specifically induce cyclic AMP production or Ca2+ release in *Drosophila melanogaster*. J Neurochem.

[B38] Maqueira B, Chatwin H, Evans PD (2005). Identification and characterization of a novel family of *Drosophila *β-adrenergic-like octopamine G-protein coupled receptors. J Neurochem.

[B39] Clyne PJ, Brotman JS, Sweeney ST, Davis G (2003). Green fluorescent protein tagging *Drosophila *proteins at their native genomic loci with small P elements. Genetics.

[B40] Poskanzer KE, Marek KW, Sweeney ST, Davis GW (2003). Synaptotagmin I is necessary for compensatory synaptic vesicle endocytosis *in vivo*. Nature.

[B41] Kiehart DP, Feghali R (1986). Cytoplasmic myosin from *Drosophila melanogaster*. J Cell Biol.

[B42] Renden RB, Broadie K (2003). Mutation and activation of Gα s similarly alters pre- and postsynaptic mechanisms modulating neurotransmission. J Neurophysiol.

[B43] Schuster CM, Ultsch A, Schloss P, Cox JA, Schmitt B, Betz H (1991). Molecular cloning of an invertebrate glutamate receptor subunit expressed in Drosophila muscle. Science.

[B44] Kronert WA, O'Donnell PT, Fieck A, Lawn A, Vigoreaux JO, Sparrow JC, Bernstein SI (1995). Defects in the *Drosophila *myosin rod permit sarcomere assembly but cause flight muscle degeneration. J mol Biol.

[B45] Stewart BA, Atwood HL, Renger JJ, Wang J, Wu CF (1994). Improved stability of *Drosophila *larval neuromuscular preparations in haemolymph-like physiological solutions. J comp Physiol A.

[B46] Huffyuv. http://neuron2.net/www.math.berkeley.edu/benrg/huffyuv.html.

[B47] AviLine. http://biolpc22.york.ac.uk/avianal/avi_line/.

[B48] Goldberger AL, Amaral LAN, Glass L, Hausdorff JM, Ivanov PC, Mark RG, Mietus JE, Moody GB, Peng CK, Stanley HE (2000). PhysioBank, PhysioToolkit, and PhysioNet : Components of a New Research Resource for Complex Physiologic Signals. Circulation.

[B49] Miller A, Demerec M (1950). The internal anatomy and histology of imago of *Drosophila melanogaster*. Biology of Drosophila.

[B50] Fly Ovary. http://biolpc22.york.ac.uk/drosophila/ovary/.

